# Improving Short- and Long-Term Genetic Gain by Accounting for Within-Family Variance in Optimal Cross-Selection

**DOI:** 10.3389/fgene.2019.01006

**Published:** 2019-10-29

**Authors:** Antoine Allier, Christina Lehermeier, Alain Charcosset, Laurence Moreau, Simon Teyssèdre

**Affiliations:** ^1^GQE-Le Moulon, INRA, Univ. Paris-Sud, CNRS, AgroParisTech, Université Paris-Saclay, Gif-sur-Yvette, France; ^2^Genetics and Analytics Unit, RAGT2n, Druelle, France

**Keywords:** genomic prediction, optimal cross-selection, usefulness criterion, parental contributions, genetic diversity, Bulmer effect

## Abstract

The implementation of genomic selection in recurrent breeding programs raises the concern that a higher inbreeding rate could compromise the long-term genetic gain. An optimized mating strategy that maximizes the performance in progeny and maintains diversity for long-term genetic gain is therefore essential. The optimal cross-selection approach aims at identifying the optimal set of crosses that maximizes the expected genetic value in the progeny under a constraint on genetic diversity in the progeny. Optimal cross-selection usually does not account for within-family selection, i.e., the fact that only a selected fraction of each family is used as parents of the next generation. In this study, we consider within-family variance accounting for linkage disequilibrium between quantitative trait loci to predict the expected mean performance and the expected genetic diversity in the selected progeny of a set of crosses. These predictions rely on the usefulness criterion parental contribution (UCPC) method. We compared UCPC-based optimal cross-selection and the optimal cross-selection approach in a long-term simulated recurrent genomic selection breeding program considering overlapping generations. UCPC-based optimal cross-selection proved to be more efficient to convert the genetic diversity into short- and long-term genetic gains than optimal cross-selection. We also showed that, using the UCPC-based optimal cross-selection, the long-term genetic gain can be increased with only a limited reduction of the short-term commercial genetic gain.

## Introduction

Successful breeding requires strategies that balance immediate genetic gain with the maintenance of population diversity to sustain long-term progress (Jannink, 2010). At each selection cycle, plant breeders are facing the choice of new parental lines and the way in which these are mated, to improve the mean population performance and generate the genetic variation on which selection will act. As breeding programs from different companies compete for short-term gain, breeders tend to use intensively the most performant individuals sometimes at the expense of genetic diversity (Rauf et al., 2010; Gerke et al., 2015; Allier et al., 2019a). The identification of the crossing plan that maximizes the performance in progeny and limits diversity reduction for long-term genetic gain is essential.

Historically, breeders used to select the best individuals based on phenotypic observations, considered as a proxy of their breeding value, i.e., the expected value of their progeny. In order to better estimate the breeding value of individuals, phenotypic selection has been complemented by pedigree-based prediction of breeding values (Henderson, 1984; Piepho et al., 2008) and more recently by genomic prediction of breeding values (Meuwissen et al., 2001), taking advantage of the availability of cheap high-density genotyping. In genomic selection (GS), a model calibrated on phenotype and genotype information of a training population is used to predict genomic estimated breeding values (GEBVs) from genome-wide marker information. A truncation selection is commonly applied on GEBVs, and the selected individuals are intercrossed to create the next generation. The interest of GS is due to the acceleration of selection progress by shortening generation interval, the increase in selection intensity, and the increase in accuracy (Hayes et al., 2010; Daetwyler et al., 2013; Heslot et al., 2015). As a consequence, compared to phenotypic selection, GS is expected to accelerate the loss of genetic diversity due to the rapid fixation of genomic regions with large effects, but also the higher probability to select individuals that are the closest to the training population and are therefore predicted more accurately (Clark et al., 2011; Pszczola et al., 2012). As a result, it has been shown in an experimental study (Rutkoski et al., 2015) and by stochastic simulations (Jannink, 2010; Lin et al., 2016) that GS increases the loss of diversity compared to phenotypic selection. Thus, the optimization of mating strategies in GS breeding programs is a critical area of theoretical and applied research.

Several approaches have been suggested to balance the short- and long-term genetic gain while selecting crosses in GS. In line with Kinghorn, (2011), Pryce et al. (2012), and Akdemir and Isidro-Sánchez (2016), the selection of a set of crosses requires two components: (i) a cross-selection index (CSI) that measures the interest of a set of crosses and (ii) an algorithm to find the set of crosses that maximizes the CSI.

The CSI may consider crosses individually; i.e., the interest of a cross does not depend on the other crosses in the selected set. In classical recurrent GS, candidates with the highest GEBVs are selected and intercrossed to maximize the expected progeny mean in the next generation. In this case, the CSI is simply the mean of parental GEBVs. However, such an approach maximizes neither the expected response to selection in the progeny, which involves genetic variance generated by Mendelian segregation within each family, nor the long-term genetic gain. Alternative measures of the interest of a cross have been proposed to account for parent complementarity, based on within cross variability and expected response to selection. Daetwyler et al. (2015) proposed the optimal haploid value (OHV) that accounts for the complementarity between parents of a cross for predefined haplotype segments. Using stochastic simulations, the authors observed that OHV selection yielded higher long-term genetic gain and preserved greater amount of genetic diversity than truncation GS. However, OHV accounts for neither the position of quantitative trait loci (QTLs) nor the linkage disequilibrium between QTLs (Lehermeier et al., 2017b; Müller et al., 2018). Schnell and Utz (1975) proposed the usefulness criterion (UC) of a cross to evaluate the expected response to selection in its progeny. The UC of a cross accounts for the progeny mean (μ) that is the mean of parental GEBVs and the progeny standard deviation (σ) the selection intensity (*i*) and the selection accuracy (*h*): *UC* = μ + *ih*σ. Zhong and Jannink (2007) proposed to predict progeny variance using estimated QTL effects, accounting for linkage between loci. Genome-wide marker effects have also been considered to predict the progeny variance with computationally intensive stochastic simulations (e.g., Mohammadi et al., 2015). Recently, an unbiased predictor of progeny variance (σ^2^) has been derived in Lehermeier et al. (2017b) for two-way crosses and extended in Allier et al. (2019b) for multiparental crosses implying up to four parents. Lehermeier et al. (2017b) observed that using UC as a CSI increased the short-term genetic gain compared to using OHV or mean parental GEBV. Similar results have been obtained by simulations by Müller et al. (2018), considering the expected maximum haploid breeding value (EMBV) that is akin to the UC for normally distributed and fully additive traits.

Alternatively, one can consider a more holistic CSI for which the interest of a cross depends on the other selected crosses. This is the case in optimal contribution selection (Wray and Goddard, 1994; Meuwissen, 1997; Woolliams et al., 2015), where a set of candidate parents is evaluated as a whole regarding the expected short-term gain and the associated risk on loosing long-term gain. Optimal contribution selection aims at identifying the optimal contributions (*c*) of candidate parents to the next generation obtained by random mating, in order to maximize the expected genetic value in the progeny (*V*) under a certain constraint on inbreeding (*D*). Optimal cross-selection, further referred as OCS, is an extension of the optimal contribution selection to deliver a crossing plan that maximizes *V* by considering additional constraints on the allocation of mates in crosses to limit *D* (Kinghorn et al., 2009; Kinghorn, 2011; Akdemir and Isidro-Sánchez, 2016; Gorjanc et al., 2018; Akdemir et al., 2018). In GS, the expected genetic value in progeny (*V*) to be maximized is the mean of parental GEBV (***a***) weighted by parental contributions ***c***, i.e ***c’a***, and the constraint on inbreeding (*D*) to be minimized is ***c***’***Kc*** with ***K*** a genomic coancestry matrix. Differential evolutionary algorithms have been proposed to obtain optimal solutions for ***c*** and the crossing plan (Storn and Price, 1997; Kinghorn et al., 2009; Kinghorn, 2011). Optimal contribution selection is commonly used in animal breeding (Woolliams et al. 2015) and is increasingly adopted in plant breeding (Akdemir and Isidro-Sánchez, 2016; De Beukelaer et al., 2017; Lin et al., 2017; Gorjanc et al., 2018; Akdemir et al., 2018).

In plant breeding, one typically has larger biparental families than in animal breeding. Especially with GS, the selection intensity within-family can be largely increased so that plant breeders capitalize much more on the segregation variance within families than animal breeders. In previous works, the genetic gain (*V*) and constraint (*D*) have been defined at the level of the progeny before within-family selection. Exceptions are the work of Shepherd and Kinghorn (1998) and Akdemir and Isidro-Sánchez (2016); Akdemir et al. (2018), who added a term to *V* accounting for within cross variance assuming linkage equilibrium between QTLs. To our knowledge, no previous study considered linkage disequilibrium (LD) between QTLs. Furthermore, as observed in historical wheat data (Fradgley et al., 2019) and using simulations in a maize context (Allier et al., 2019b), within-family selection also affects the effective contribution of parents to the next generation. This likely biases the prediction of inbreeding/diversity in the next generation, which to our knowledge has not been considered in previous studies.

**Figure 1:**
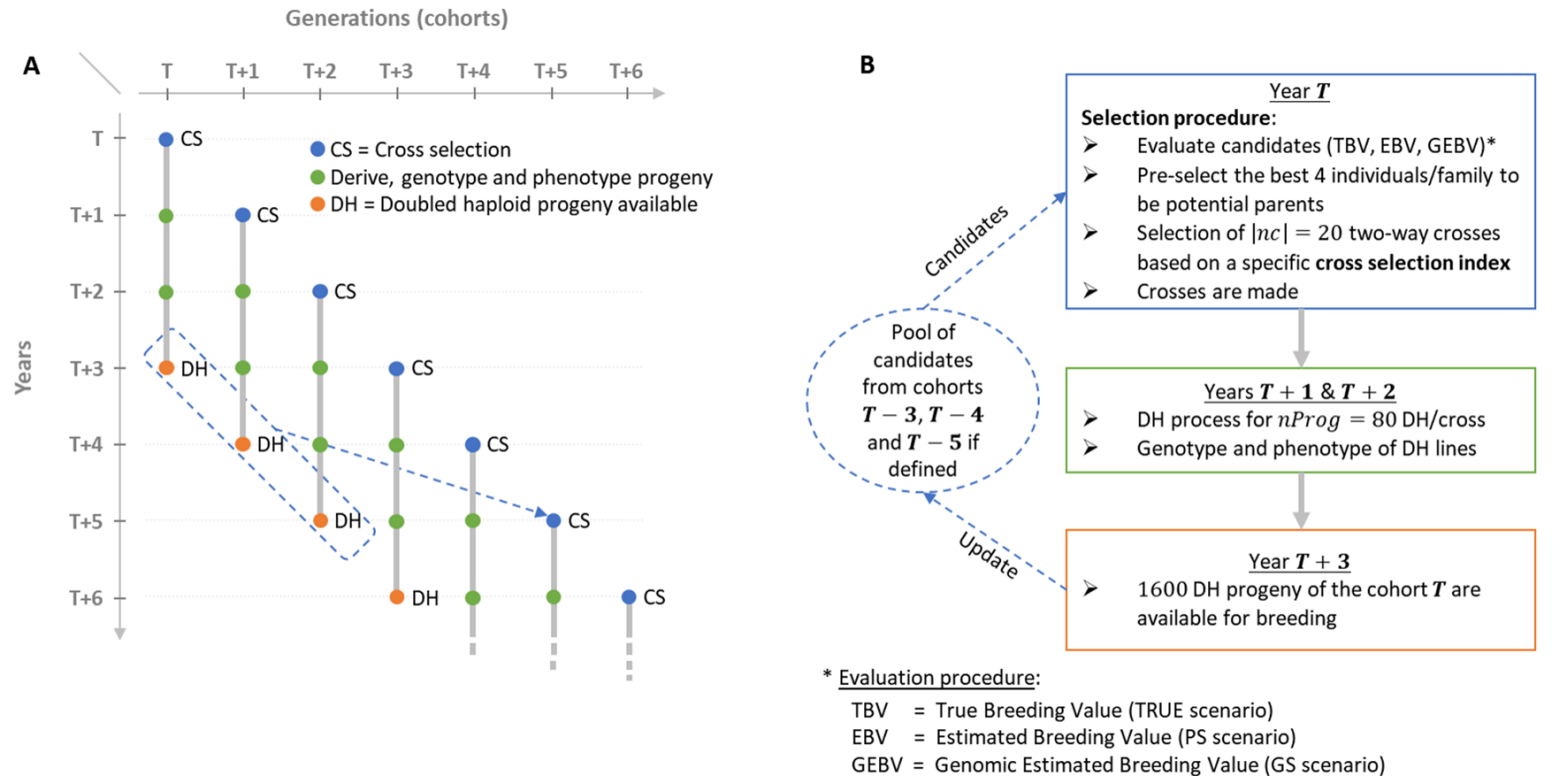
Schematic view of the simulated breeding program. **(A)** Overall view of the breeding program and overlapping cohorts. **(B)** Life cycle of a given post burn-in cohort *T* depending on the scenario considered (TRUE with 1,000 known QTL effects, PS in absence of genomic information or GS with 2,000 noncausal SNPs estimated effects).

In this study, we propose to adjust *V* and *D* terms so that within-family selection of the candidate parents for the next generation is accounted for. We propose to use the usefulness criterion parental contribution (UCPC) approach (Allier et al., 2019b) that enables to predict the expected mean performance of the selected fraction of progeny and to predict the contribution of parents to the selected fraction of progeny. We compared our OCS strategy based on UCPC with other cross-selection strategies, in a long-term simulated recurrent GS breeding program involving overlapping generations (**Figure 1A**). Our objectives were to demonstrate (1) the interest of UCPC to predict the genetic diversity in the selected fraction of progeny and (2) the interest of accounting for within-family selection in OCS for both short- and long-term genetic gains.

## Materials and Methods

### Simulated Breeding Program

#### Breeding Program

We simulated a breeding program to compare the effect of different CSIs on short- and long-term genetic gain in a realistic breeding context considering overlapping and connected generations (i.e., cohorts) and the use of doubled haploid (DH) technology to derive progeny (**Figure 1A**). We considered that the process to derive DH progeny from a cross and to phenotype and genotype DH lines takes 3 years. Furthermore, we considered as candidate parents of a cohort *T* the selected fraction of DH progeny of the three last available cohorts, i.e., *T*-3, *T*-4 and *T*-5 (**Figures 1A, B**).

Each simulation replicate started from a population of 40 founders sampled among 57 Iodent maize genotypes from the Amaizing project (Rio et al., 2019; Allier et al., 2019b). We sampled 1,000 biallelic QTLs among the 40,478 high-quality single-nucleotide polymorphisms (SNPs) from the Illumina MaizeSNP50 BeadChip (Ganal et al. 2011), with consensus genetic positions from Giraud et al. (2014). The sampling process obeyed two constrains: a QTL minor allele frequency ≥ 0.2 and a distance between two consecutive QTLs ≥ 0.2 cM. Each QTL was assigned an additive effect sampled from a Gaussian distribution with a mean of zero and a variance of 0.05, and the favorable allele was attributed at random to one of the two SNP alleles.

We initiated a virtual breeding program starting from the founder genotypes with a burn-in period of 20 years that mimicked recurrent phenotypic selection. Burn-in started by randomly crossing the 40 founders into 20 biparental families, i.e., two-way crosses, during the first 3 years to initiate three overlapping cohorts. In each cohort, 80 DH progeny genotypes per cross were simulated. Phenotypes were simulated considering the genotype at QTLs, an error variance corresponding to a trait repeatability of 0.4 in the founder population and no genotype by environment interactions. For phenotyping, every individual was evaluated in four environments in 1 year. Since no secondary trait was considered and sufficient seed production for extensive progeny testing was assumed, we simulated a unique within-family selection of the 5% best progeny (i.e., 4 DHs) that is a common selection intensity in maize breeding. During burn-in, we first considered within-family phenotypic selection and then used the 50 DHs with the largest phenotypic mean as potential parents of the next cohort. These were randomly mated, i.e., without any constraint on parental contributions, to generate 20 biparental families of 80 DH lines. After 20 years of burn-in, this created extensive linkage disequilibrium as often observed in elite plant breeding programs (e.g., Van Inghelandt et al., 2011). We then compared different CSIs for 60 years of recurrent GS using DH technology (**Figure 1**). As in burn-in, each cohort *T* was generated by 20 two-way crosses (|*nc|*=20) of 80 DH progeny each (*nProg* = 80). Candidate parents of cohort *T* were selected from the available DH of the three cohorts: *T*=3, *T*-4, and *T*-5 (**Figures 1A, B**). Per family, the 4 DH lines (i.e., 5%) with the largest breeding values, detailed in “Evaluation scenario” section, were considered as potential parents, yielding 4 DH lines/family × 20 families/cohort × 3 cohorts = 240 potential parents. Considering these *N* = 240 potential parents, *N*(*N*-1)/2 = 28,680 two-way crosses are possible. The set of |*nc*| = 20 two-way crosses among these 28,680 candidate crosses was defined using different CSI detailed in the following sections. This simulated scheme yielded overlapping and connected cohorts as it is standard in practical plant breeding (**Figure 1A**). A detailed description of the simulated breeding program and the material is provided in **Supplementary Material (File S1)**.

#### Evaluation Scenarios

We considered different scenarios for genome-wide marker effects and progeny evaluation. In order to eliminate the uncertainty caused by the estimation of marker effects, we first compared several CSI assuming that we have access to the positions and effects of the 1,000 QTLs (referred to as TRUE scenario). For a representative subset of the CSI showing differentiated results in the TRUE scenario, we also considered a more realistic scenario where the effects of QTLs are unknown and selection was based on the effects of 2,000 noncausal SNPs randomly sampled over the genome. In this scenario, marker effects were obtained by back-solving (Wang et al., 2012) a G-BLUP model fitted using blupf-90 AI-REML solver (Misztal, 2008). This scenario was referred to as GS scenario, and marker effects used to predict the CSI were estimated every year with all candidate parents that were phenotyped and genotyped. The progeny were selected on their GEBV considering their phenotypes and their genotypes at noncausal SNPs. As a benchmark, we also considered a phenotypic selection scenario where progeny were selected based on their phenotypic mean (PS scenario). For details on the evaluation models, see **Supplementary Material (File S1)**. In the following, for sake of clarity, we present the different cross-selection strategies considering selection based on known QTL effects and positions (TRUE scenario). In GS scenario, QTL effects and positions were replaced by estimated marker effects and positions.

### Cross-Selection Strategies

#### Optimal Cross-Selection Not Accounting for Within-Family Selection

Considering *N* homozygote candidate parents, *N*(*N*-1)/2 two-way crosses are possible. We define a crossing plan ***nc*** as a set of |*nc*| crosses out of possible two-way crosses, giving the index of selected crosses, i.e., with the *i^th^* element *nc*(*i*)∈[1,*N*(*N*-1)/2]. The (*N* × 1) dimensional vector of candidate parents contributions ***c*** is defined as

(1) $$c=\frac{1}{\left|\mathrm{nc}\right|}(Z_{1}c_{1}+\;Z_{2}c_{2})\;,$$

where ***Z***_1_ (respectively ***Z***_2_) is a (*N*× |*nc*|) dimensional design matrix that links each *N* candidate parent to the first (respectively second) parent in the set of crosses ***nc***, ***c*_1_** (respectively, ***c***_2_) is a (|*nc*|× 1) dimensional vector containing the contributions of the first (respectively, second) parent to progeny, i.e., a vector of 0.5 when assuming no selection within crosses.

The (*N* × 1) dimensional vector of candidate parents true breeding values is ***a*** = ***X***β*_T_* where ***X*** = (*x*_1_,…,***x****_N_*)’ is the (*N* ×*m*) dimensional matrix of known parental genotypes at *m* biallelic QTLs, where ***x****_p_* denotes the (*m* × 1) dimensional genotype vector of parent *p*∈[1,*N*] with the *j^th^* element coded as 1 or −1 for the genotypes AA or aa at QTL *j*. β*_T_* is the (*m*× 1) dimensional vector of known additive QTL effects for the quantitative agronomic performance trait considered. The genetic gain *V*(***nc***) for this set of two-way crosses is defined as the expected mean performance in the DH progeny:

(2) V(nc)=c'a .

We define the constraint on diversity (*D*) as the mean expected genetic diversity in DH progeny (He,Nei, 1973):

(3) $$D\left(\mathrm{nc}\right)=1-\;c^{\prime }Kc\;,$$

where $\;K=\frac{1}{2}\left(\frac{1}{m}XX^{\prime }+1\right)$ is the (*N*× *N*) dimensional identity by state (IBS) coancestry matrix between the *N* candidates. **Supplementary Material (File S2)** details the relationship between the IBS coancestry among parents (***K***), the parental contributions to progeny (***c***) and the mean expected heterozygosity in progeny $\mathrm{He}=\frac{1}{m}\overset{m}{\underset{j=1}{\sum }}2p_{j}\left(1-p_{j}\right)$ where *p_j_* the frequency of the genotypes AA at QTL *j* in the progeny.

#### Accounting for Within-Family Selection in OCS

In the OCS, as defined above, the progeny derived from the ***nc*** crosses are all expected to contribute to the next generation. We propose to consider *V*(***nc***) and *D*(***nc***) terms accounting for the fact that only a selected fraction of each family will be candidate for the next generation (e.g., 5% per family in our simulation study). For this, we apply the UCPC approach proposed by Allier et al. (2019b) for two-way crosses and extend its use to evaluate the interest of a set ***nc*** of two-way crosses after selection in progeny.

##### UCPC for Two-Way Crosses

Two inbred lines *P*_1_ and *P*_2_ are considered as parental lines for a candidate cross *P*_1_× *P*_2_ and (***x***_1_, ***x***_2_)’ denotes their genotyping matrix. Following Lehermeier et al. (2017b), the DH progeny mean and progeny variance of the performance in the progeny before selection can be computed as follows:

(4a) $$\mu_{T}=0.5\;(x{'}_{1}\beta_{T}+x{'}_{2}\beta_{T})\;,$$

(4b) $$\sigma_{T}^{2}=\beta_{T}^{'}\;\Sigma \;\beta_{T}\;,$$

where ***x***_1_, ***x***_2_ and β*_T_* were defined previously, and Σ is the (*m*×*m*) -dimensional variance covariance matrix of QTL genotypes in DH progeny defined in Lehermeier et al. (2017b).

To follow parental contributions, we consider *P*_1_ parental contribution as a normally distributed trait (Allier et al., 2019b). As we only consider two-way crosses and biallelic QTLs, we can simplify for computational reasons the formulas by using IBS parental contributions computed for polymorphic QTLs between *P*_1_ and *P*_2_ instead of using identity-by-descent parental contributions (Allier et al., 2019b). We define the (*m*×1) -dimensional vector β*_C_*_1_ to follow *P*_1_ genome contribution at QTLs as $\beta_{C1}=\frac{x_{1}-x_{2}}{\left(x_{1}-x_{2}\right)'\left(x_{1}-x_{2}\right)}$. We compute the mean of *P*_1_ contribution in the progeny before selection *μ_C_*_1_=0.5(***x***’_1_β*_C_*_1_+***x***’_2_β*_C_*_1_+1). The progeny variance $\sigma_{C1}^{2}$ for *P*_1_ contribution in the progeny before selection is computed using Eq. 4b by replacing β*_T_* by β*_C_*_1_ The progeny mean for *P*_2_ contribution is then defined as *μ_C_*_2_ = 1-*μ_C_*_1_.

Following Allier et al. (2019b), we compute the covariance between the performance and *P*_1_ contribution in progeny as follows:

(5) $$\sigma_{T,\;\;C1\;}=\beta {'}_{T}\Sigma \;\beta_{C1}\;.$$

The expected mean performance of the selected fraction of progeny, i.e., UC (Schnell and Utz, 1975), of the cross *P*_1_×*P*_2_ is as follows:

(6) $$UC^{\left(i\right)}=\mu_{T}+ih\sigma_{T}\;,$$

where *i* is the within-family selection intensity, and the exponent (*i*) in *UC* expresses the dependency of *UC* on the selection intensity *i*. We considered a selection accuracy *h*=1 as in Zhong and Jannink (2007), which holds when selecting on true breeding values in TRUE scenario. As discussed further, we also considered *h* = 1 when selecting crosses based on UCPC in GS scenario. The correlated responses to selection on *P*_1_ and *P*_2_ genome contributions in the selected fraction of progeny are as follows (Falconer and Mackay, 1996):

(7) $$c_{1}^{\left(i\right)}=\mu_{C1}+i\frac{\sigma_{T,C1}}{\sigma_{T}}\text{ and }c_{2}^{\left(i\right)}=1-c_{1}^{\left(i\right)}\;.$$

##### Cross-Selection Based on UCPC

Accounting for within-family selection intensity *i*, the genetic gain term *V*^(^*^i^*^)^(***nc***) for a set of two-way crosses ***nc*** is defined as the expected performance in the selected fraction of progeny:

(8) $$V^{\left(i\right)}\left(\mathrm{nc}\right)=\frac{1}{\left|\mathrm{nc}\right|}\;\sum_{j\in \mathrm{nc}}UC^{\left(i\right)}(j).$$

The constraint on diversity *D*^(^*^i^*^)^(***nc***) in the selected progeny is defined as follows:

(9) D(i)(nc)=1− c(i)'Kc(i) ,

where ***c***^(^*^i^*^)^ is defined like ***c*** in Eq. 1 but accounting for within-family selection by replacing the ante-selection parental contributions ***c***_1_ and ***c***_2_ by the post-selection parental contributions $c_{1\;}^{\left(i\right)}$ and $c_{2\;}^{\left(i\right)}$ (Eq. 7), respectively. Note that considering the absence of selection in progeny, i.e., *i* = 0, yields *V*^(^*^i^* ^= 0)^(***nc***) being the mean of parent breeding values (Eq. 2) and *D*^(^*^i^* ^= 0)^(***nc***) being the expected diversity in progeny before selection (Eq. 3), which is equivalent to optimal cross-selection as proposed by Gorjanc et al. (2018). The R code (R Core Team, 2017) to evaluate a set of crosses as presented in the UCPC-based optimal cross-selection is provided in **Supplementary Material (File S3)**.

#### Multiobjective Optimization Framework 

**Figure 2:**
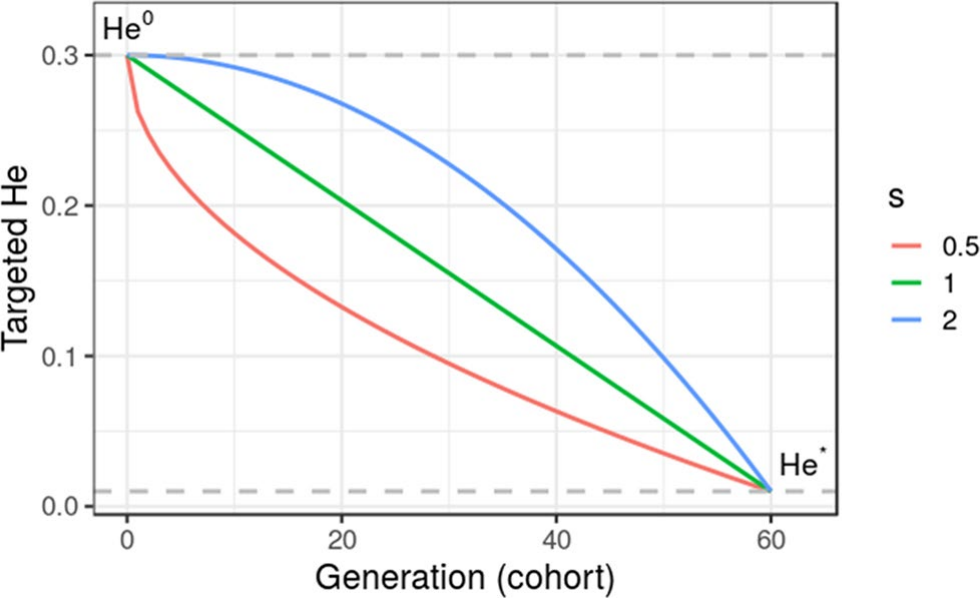
Targeted diversity trajectories for three different shape parameters (s = 1, linear trajectory; s = 2, quadratic trajectory; and s = 0.5, inverse quadratic trajectory) for fixed initial diversity (He^0^ = 0.3) at generation 0 and targeted diversity (He* = 0.01) at generation 60 (t* = 60). We considered in this study only linear trajectories (s = 1).

In practice, one does not evaluate only one set of crosses but several ones in order to find the optimal set of crosses to reach a specified target that is a function of *V*^(^*^i^*^)^(***nc***) and *D*^(^*^i^*^)^(***nc***). We use the ε-constraint method (Haimes et al., 1971;Gorjanc and Hickey, 2018) to solve the multiobjective optimization problem:

(10) $$\begin{array}{l} \underset{\mathrm{nc}}{\max}\;V^{\left(i\right)}\left(\mathrm{nc}\right)\\ \text{with}\;D^{\left(i\right)}\left(\mathrm{nc}\right)\geq \;\mathrm{He}\left(t\right) \end{array}\;,$$

where *He*(*t*), ∀*t*∈[0,t^*^] is the minimal diversity constraint at time *t*. A differential evolutionary (DE) algorithm was implemented to find the set of ***nc*** crosses that is a Pareto-optimal solution of Eq. 10 (Storn and Price, 1997; Kinghorn et al., 2009; Kinghorn, 2011). DE is an optimization process inspired by natural selection. It started from an initial population of 7,170 random candidate solutions that are improved during 1,000 iterations through mutation (random changes in candidate solutions), recombination (exchanges between candidate solutions), and selection (every iteration a candidate solution was replaced by its mutated and recombined version if superior). The direct consideration of *He*(*t*) in the optimization allows to control the decrease in genetic diversity similarly to what was suggested for controlling inbreeding rate in animal breeding (Woolliams et al., 1998, Woolliams et al., 2015). The loss of diversity along time is controlled by the targeted diversity trajectory, i.e., *He*(*t*), ∀*t*∈[0,t^*^], where t^*^∈ℕ^*^ is the time horizon when the genetic diversity *He*(t^*^) = He^*^ should be reached. In this study, *He*(*t*) is defined as follows:

(11) $$\mathrm{He}\left(t\right)=\{\begin{array}{c} He^{0}+{\left(\frac{t}{t^{*}}\right)}^{s}\left(He^{*}-He^{0}\right),\;\forall \;t\in \;〚0,t^{*}〛\;\;\\ He^{*},\;\forall \;t>t^{*} \end{array},$$

where *He*^0^ is the initial diversity at *t* = 0, and *s* is a shape parameter with *s* = 1 for a linear trajectory. **Figure 2** gives an illustration of alternative trajectories that can be defined using Eq. 11.

#### Cross-Selection Indices 

We considered different cross-selection approaches varying in the within-family selection intensity (*i*) in *V*^(^*^i^*^)^(***nc***), *D*^(^*^i^*^)^(***nc***) (Eq. 10) and in the targeted diversity trajectory *He*(*t*) (Eq. 11). We first considered as a benchmark the absence of constraint *D*^(^*^i^*^)^(***nc***), i.e., *He*(*t*) = 0, ∀*t*. We defined two alternative CSIs PM (parental mean) and UC, respectively considering *V*^(^*^i^* ^= 0)^(***nc***) and *V*^(^*^i^* ^= 2.06)^(***nc***), with *i* = 2.06 corresponding to the selection of the 5% most performant progeny per family. PM is equivalent to cross the best candidates together without accounting for within cross variance, while UC is defined as crossing candidates based on the expected mean performance of the 5% selected fraction of progeny. Note that the absence of constraint on diversity also means the absence of constraint on parental contributions. To compare optimal cross-selection accounting or not for within-family selection, we considered three linear diversity trajectories (Eq. 11) with He^*^ = {0.01, 0.10, 0.15} that should be reached in t^*^ = 60 years. We defined the OCS methods, further referred to as OCS-He*, with *V*^(^*^i^* ^= 0)^(***nc***) and *D*^(^*^i^* ^= 0)^(***nc***). We defined the UCPC cross-selection methods, further referred to as UCPC-He*, with *V*^(^*^i^* ^= 2.06)^(***nc***) and *D*^(^*^i^* ^= 2.06)^(***nc***). The eight CSIs considered are summarized in **Table 1**.

**Table 1:** Summary of tested cross-selection indices (CSI) in TRUE scenario defined for a set of crosses ***nc*** depending on the within-family selection intensity *i*.

Cross-selection index (CSI)	Gain term	Diversity term
PM	*V*^(^*^i^* ^= 0)^(***nc***)	–
OCS-He* (3 different He*)	*V*^(^*^i^* ^= 0)^(***nc***)	*D*^(^*^i^*^= 0)^(***nc***)
UC	*V*^(^*^i^* ^= 2.06)^(***nc***)	–
UCPC-He* (3 different He*)	*V*^(^*^i^* ^= 2.06)^(***nc***)	*D*^(^*^i^* ^= 2.06)^(***nc***)

He* = {0.15; 0.10; 0.01} to be reached linearly (s = 1) at the end of simulation (t^*^ = 60 years). V^(i = 0)^(**nc**) is the averaged parental mean (PM) of crosses in **nc** and V^(i = 2.06)^(**nc**) is the averaged usefulness criterion (UC) of crosses in **nc** considering a within-family selection intensity of 2.06. D^(i = 0)^(**nc**) and D^(i = 2.06)^(**nc**) are the expected genetic diversity in the progeny before and after within-family selection, respectively.

### Simulation 1: Interest of UCPC to Predict the Diversity in the Selected Fraction of Progeny

Simulation 1 aimed at evaluating the interest to account for the effect of selection on parental contributions, i.e., post-selection parental contributions (using UCPC), compared to ignore selection, i.e., ante-selection parental contributions (similarly as in OCS), to predict the genetic diversity (He) in the selected fraction of progeny of a set of 20 crosses (using Eqs. 9 and 3, respectively). We considered a within-family selection intensity corresponding to selecting the 5% most performant progeny. We used the same genotypes, genetic map, and known QTL effects as for the first simulation replicate of the PM CSI in the TRUE scenario (**Table 1**). We extracted the simulated genotypes of 240 DH candidate parents of the first post burn-in cohort (further referred as E1) and of 240 DH candidate parents of the 20th post burn-in cohort (further referred as E2). Due to the selection process, E1 showed a higher diversity and lower performance compared to E2. We randomly generated 300 sets of 20 two-way crosses: 100 sets of intrageneration E1 crosses (E1 × E1), 100 sets of intrageneration E2 crosses (E2 × E2), and 100 sets of intergeneration and intrageneration crosses randomly sampled (E1 × E2, E1 × E1, E2 × E2). We derived 80 DH progeny per cross and predicted the ante- and post-selection parental contributions to evaluate the post-selection genetic diversity (He) for each set of crosses. We estimated the empirical post-selection diversity for each set of crosses and compared predicted and empirical values considering the mean prediction error as the mean of the difference between predicted He and empirical post-selection He, and the prediction accuracy as the squared correlation between predicted He and empirical post-selection He.

### Simulation 2: Comparison of Different Csis

We ran 10 independent simulation replicates of all eight CSI summarized in **Table 1** for 60 years post burn-in considering known effects at the 1,000 QTLs (TRUE scenario). We also compared in 10 independent simulation replicates the CSI: PM, UC, OCS-He* and UCPC-He* with He* = 0.01 considering estimated marker effect at the 2,000 SNPs (GS scenario) and PM based only on phenotypic evaluation (PS scenario). We followed several variables on the 80 DH progeny/family × 20 crosses realized every year. At each cohort *T*∈[0,60] with *T* = 0 co rresponding to the last burn-in cohort, we computed the additive genetic variance as the variance of the 1,600 DH progeny true breeding values (TBVs): $\sigma_{A}^{2}\left(T\right)=var\left(TBV\left(T\right)\right)$. We followed the mean genetic merit of all progeny *μ*(*T*) = mean(*TBV*(*T*)) and of the 10 most performant progeny *μ*_10_(ဃ*Tဃ*)= mean(max(*TBV*(*T*))) as a proxy of realized performance that could be achieved at a commercial level by releasing these lines as varieties. Then, we centered and scaled the two genetic merits to obtain realized cumulative genetic gains in units of genetic standard deviation at the end of the burn-in (*T* = 0), at the whole progeny level $G(T)=\left(\mu (T)-\mu (0)\right)/\sqrt{\sigma_{A}^{2}(0)}\;$ and at the commercial level $G_{10}(T)=\left(\mu_{10}(T)-\mu (0)\right)/\sqrt{\sigma_{A}^{2}(0)}$.

The interest of long-term genetic gain relies on the ability to breed at long term, which depends on the short-term economic success of breeding. Following this rationale, we penalized strategies that compromised the short-term commercial genetic gain using the discounted cumulative gain following Dekkers et al. (1995) and Chakraborty et al. (2002). In practice, we computed the weighted sum of the commercial gain value in each generation $\overset{60}{\underset{T=1}{\sum }}w_{T}\;G_{10}\left(T\right)$, where the discounted weights *w_T_*=1/(1+ρ)*^T^*,∀*T*∈[1,60] were scaled to have $\overset{60}{\underset{T=1}{\sum }}w_{T}\;=1$ and ρ is the interest rate per generation. The discounted weights measure how much breeders will care about future genetic gain compared to today’s genetic gain, also referred as the “net present value” of long-term gain in finance. For ρ = 0, the weights were *w_T_*_∈[1,60]_ = 1/60; i.e., the same importance was given to all cohorts. We compared different values of ρ and reported results for ρ = 0, ρ = 0.04 giving approximatively seven times more weight to short-term gain (after 10 years) compared to long-term gain (after 60 years) and ρ = 0.2 giving nearly no weight to gain after 30 years of breeding.

We also measured the additive genic variance at QTLs $\sigma_{a}^{2}(T)=\sum_{j=1}^{m}4\;p_{j}(T)\left(1-p_{j}(T)\right)\beta_{j}^{2}$, the mean expected heterozygosity at QTLs (He, Nei, 1973) $\mathrm{He}\;(T)=m^{-1}\sum_{j=1}^{m}2\;p_{j}(T)\left(1-p_{j\;}(T)\right)$, and the number of QTLs where the favorable allele was fixed or lost in the progeny, with *p_j_*(*T*) the allele frequency at QTL *j*∈[1,*m*] in the 1,600 DH progeny and β*_j_* the additive effect of the QTL *j*. In addition, we considered the ratio of additive genetic over genic variance $\sigma_{A}^{2}/\sigma_{a}^{2}$. which provides an estimate of the amount of additive genic variance captured by negative covariances between QTLs, known as the Bulmer effect under directional selection (Bulmer, 1971, Bulmer, 1980; Lynch and Walsh, 1999). All these variables were further averaged on the 10 simulation replicates, and the standard error divided by the square root of the number of replicates is reported.

## Results

### Simulation 1

**Figure 3:**
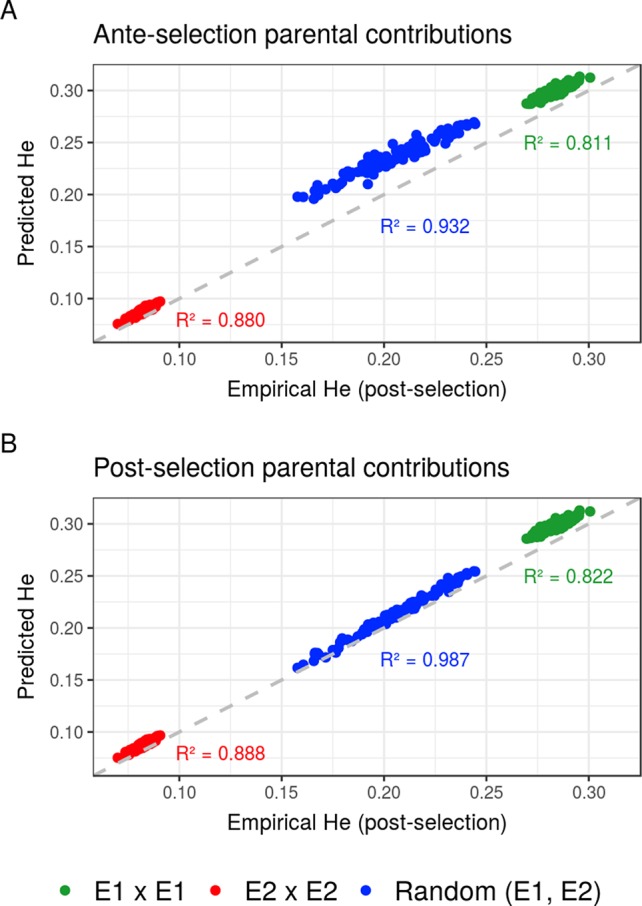
Squared correlations (*R*²) between predicted genetic diversity (He) and empirical He in the selected fraction of progeny of a set of 20 biparental crosses in the TRUE scenario considering **(A)** ante-selection parental contributions or **(B)** post-selection parental contributions to predict He. In total, 100 sets of each three types of crosses (intrageneration: E1xE1 and E2xE2 or randomly intragenerations and intergenerations): random (E1, E2) are shown, and the squared correlations between predicted and empirical post-selection He are given in the corresponding color.

**Figure 4:**
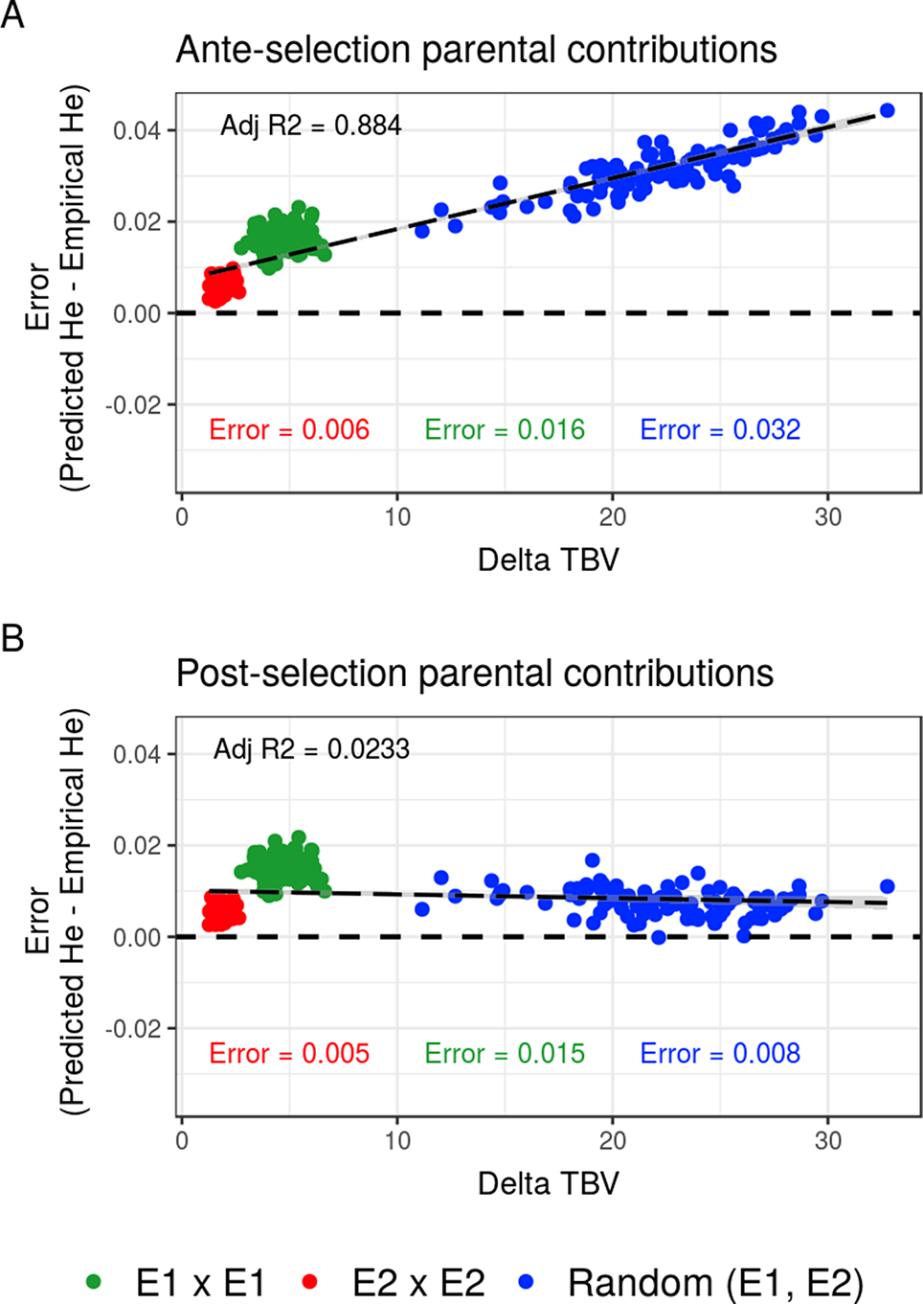
Mean prediction error (predicted − empirical) of predicting the genetic diversity (He) in the selected fraction of progeny of a set of 20 biparental crosses in the TRUE scenario depending on the mean difference of performance between parents (Delta true breeding value TBV). Mean prediction error is measured as the predicted He − empirical post-selection He, considering **(A)** ante-selection parental contributions or **(B)** post-selection parental contributions to predict He. In total, 100 sets of each three types of crosses (intrageneration: E1 × E1 and E2 × E2 or randomly intra and inter-generations): random (E1, E2) are shown, and the averaged errors are given in the corresponding color.

Compared to the usual approach that ignores the effect of selection on parental contributions, accounting for the effect of within-family selection increased the squared correlation (*R*²) between predicted genetic diversity and genetic diversity in the selected fraction of progeny (**Figures 3A, B**) for all three types of crosses. The squared correlation between predicted genetic diversity and post-selection genetic diversity for intrageneration crosses was only slightly increased (E1 × E1: from 0.811 to 0.822 and E2 × E2: from 0.880 to 0.888), while the squared correlation for sets of crosses involving also intergeneration crosses showed a larger increase (from 0.937 to 0.987) (**Figures 3A, B**). Using post-selection parental contributions instead of ante-selection parental contributions also reduced the mean prediction error of He (predicted − empirical He) (**Figures 4A, B**) for all three types of crosses. The mean prediction error for intrageneration crosses was only slightly reduced (E1 × E1: from 0.006 to 0.005 and E2 × E2: from 0.016 to 0.015), while the mean prediction error for sets involving intergeneration crosses was more reduced (from 0.032 to 0.008) (**Figures 4A, B**). The mean prediction error of He was reduced but still positive when considering post-selection parental contributions, which means that the genetic diversity in the selected fraction of progeny remains overestimated. Note that the ante-selection contributions predicted well the empirical genetic diversity before selection for all three types of crosses (mean prediction error = 0.000 and *R*² > 0.992, results not shown).

### Simulation 2 

#### Interest of UC Over PM

**Figure 5:**
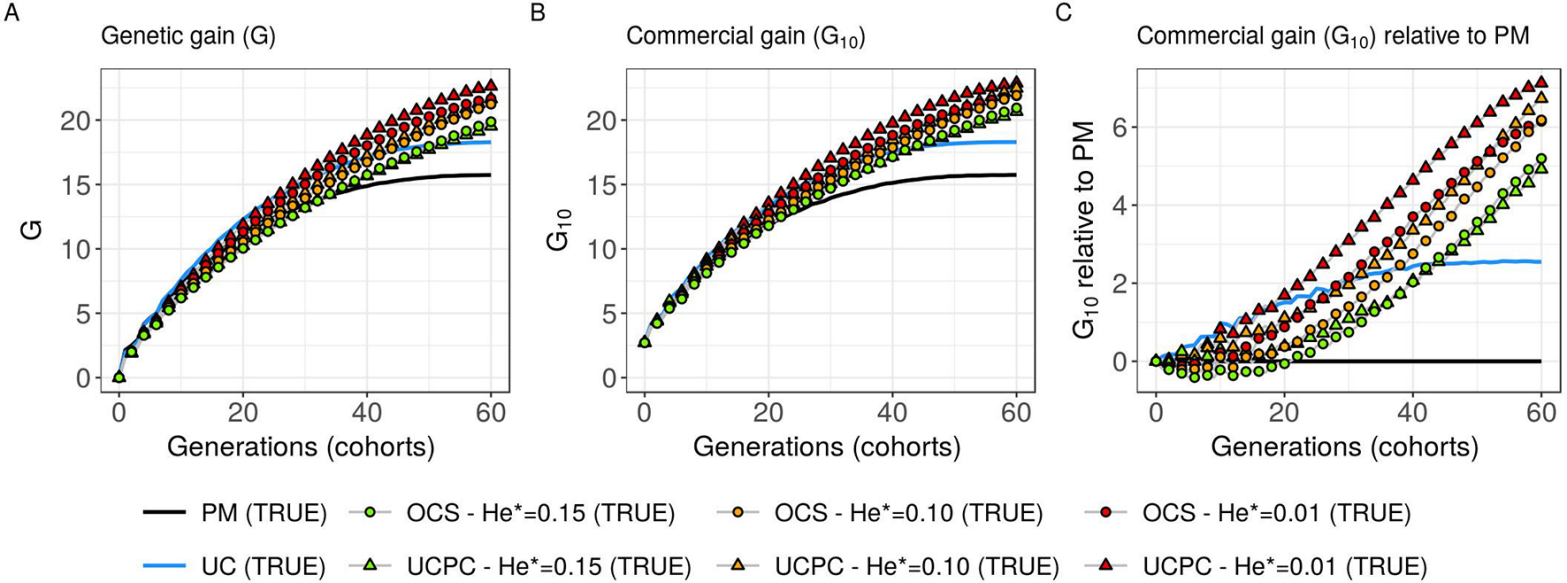
Genetic gains for different cross-selection indices in the TRUE scenario (PM: parental mean, UC: usefulness criterion, OCS-He*: optimal cross-selection and UCPC-He*: UCPC-based optimal cross-selection) according to the generations. **(A)** Genetic gain (G) measured as the mean of the whole progeny, **(B)** commercial genetic gain (*G*_10_) measured as the mean of the 10 best progeny, and **(C)** *G*_10_ relative to selection based on parental mean (PM).

**Figure 6:**
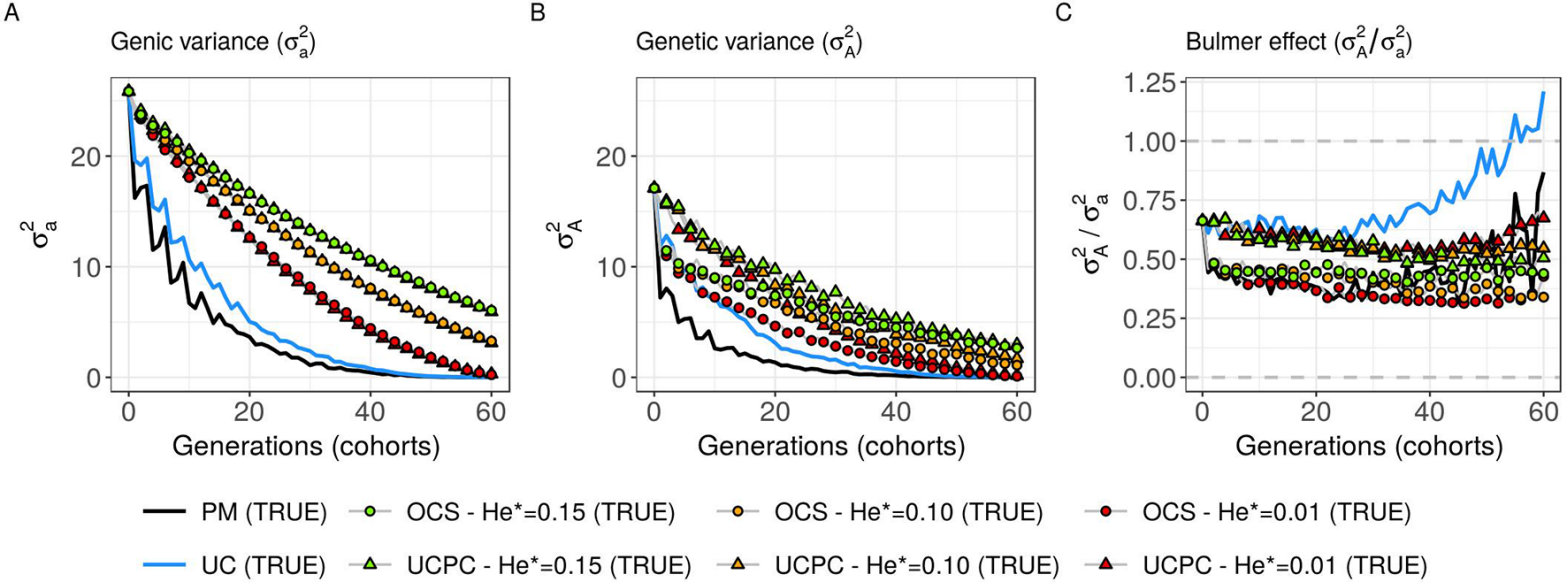
Genetic and genic additive variances for different cross-selection indices in the TRUE scenario (PM: parental mean, UC: usefulness criterion, OCS-He*: optimal cross-selection, and UCPC-He*: UCPC-based optimal cross-selection) according to the generations. **(A)** Additive genic variance ($\sigma_{a}^{2}$) measured on the whole progeny, **(B)** additive genetic variance ($\sigma_{A}^{2}$) measured on the whole progeny, and **(C)** ratio of genetic over genic variance ($\sigma_{A}^{2}/\sigma_{a}^{2}$) reflecting the Bulmer effect.

**Figure 7:**
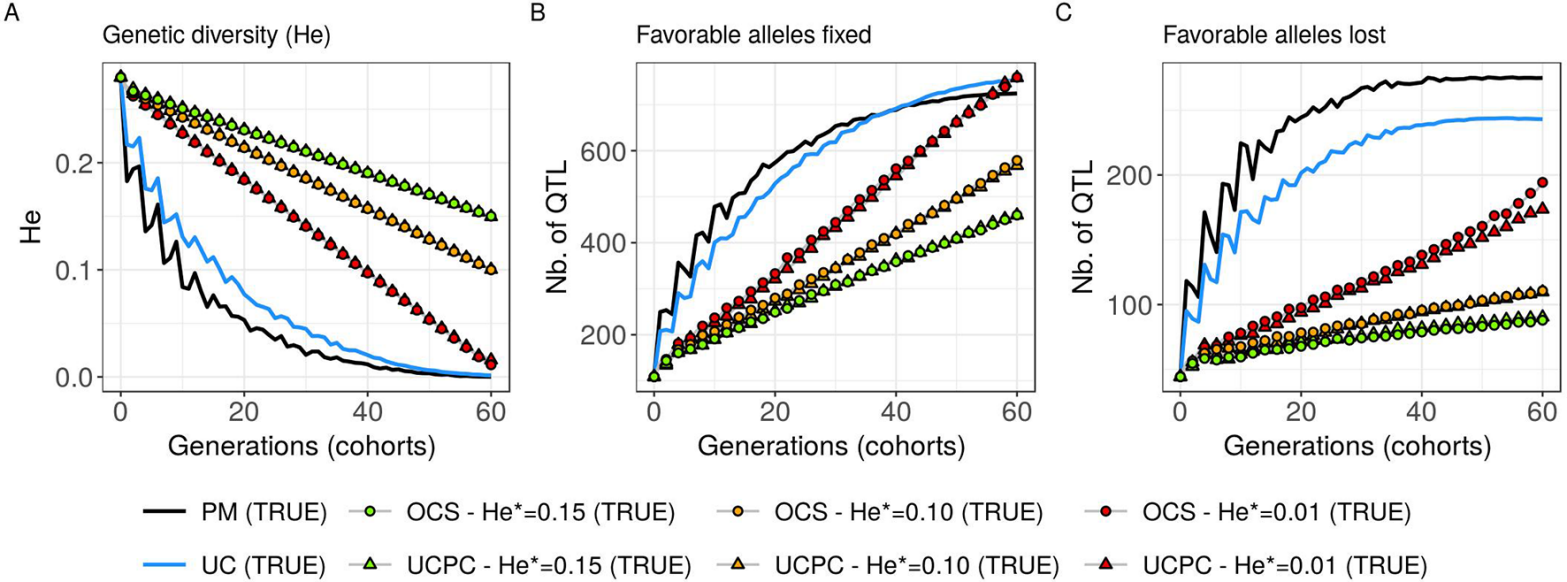
Genetic diversity at QTLs for different cross-selection indices in the TRUE scenario (PM: parental mean, UC: usefulness criterion, OCS-He*: optimal cross-selection, and UCPC-He*: UCPC-based optimal cross-selection) according to the generations. **(A)** Genetic diversity at QTLs in the whole progeny (*He*), **(B)** number of QTLs where the favorable allele is fixed in the whole progeny, and **(C)** number of QTLs where the favorable allele is lost in the whole progeny.

Considering known QTL effects (TRUE scenario), we observed that UC yielded significantly higher short- and long-term genetic gain at commercial level (*G*_10_) than PM (on average, *G*_10_ = 9.316 [±0.208] compared to 8.338 [±0.195] 10 years post burn-in and *G*_10_ = 18.293 [±0.516] compared to 15.744 [±0.449] 60 years post burn-in; **Figures 5B, C**; **Supplementary Material [Table S1 File S4]**). When considering the whole progeny mean performance (*G*), PM nonsignificantly outperformed UC for the first 5 years (on average, G = 4.647 [±0.174] compared to 4.633 [±0.138] 5 years post burn-in), and after 5 years, UC significantly outperformed PM (on average, *G* = 7.620 [±0.158] compared to 7.197 [±0.199] 10 years post burn-in) [**Figure 5A**, **Supplementary Material (Table S1 File S4)**]. UC showed higher genic ($\sigma_{a}^{2}$ ) and genetic ($\sigma_{A}^{2}$) additive variances than PM (**Figures 6A, B**), but both yielded a genic and genetic variance near zero after 60 years of breeding. The genetic over genic variance ratio($\sigma_{A}^{2}/\sigma_{a}^{2}$) was also higher for UC compared to PM (**Figure 6C**). The evolution of genetic diversity (He) along years followed the same tendency as the genic variance (**Figure 7A**, **Figure 6A**). UC fixed more favorable alleles at QTLs after 60 years (**Figure 7B**) and lost less favorable alleles at QTLs than PM in all 10 simulation replicates, with an average of 243.1 (±4.547) QTLs where the favorable allele was lost compared to 274.9 (±4.283) QTLs for PM [**Figure 7C**; **Supplementary Material (Table S1 File S4)]**.

#### Targeted Diversity Trajectory

Considering known QTL effects (TRUE scenario), the tested optimal cross-selection methods OCS-He* and UCPC-He* showed lower short-term genetic gain at the whole progeny level (*G*; **Figure 5A**) and at the commercial level (*G*_10_; **Figures 5B, C**) but significantly higher long-term genetic gains than UC at 60 years **Supplementary Material (Table S1 File S4)**. The lower the targeted diversity He*, the higher the short-term and midterm genetic gain at both whole progeny (*G*; **Figure 5A**) and commercial (*G*_10_; **Figures 5B, C**) levels. The higher the targeted diversity He*, the higher the long-term genetic gain except for OCS-He* = 0.10 and OCS-He* = 0.01 that performed similarly after 60 years (on average, *G*_10_ = 21.925 [±0.532] and 21.892 [±0.525]; **Figure 5B**, **Supplementary Material [Table S1 File S4]**). The highest targeted diversity (He* = 0.15) showed a strong penalty at the short term and midterm, while the intermediate targeted diversity (He* = 0.10) showed a lower penalty at the short term and midterm compared to the lowest targeted diversity (He* = 0.01) (**Figures 5A–C**).

For all targeted diversities and all simulation replicates, accounting for within-family selection (UCPC-He*) yielded a significantly higher short-term commercial genetic gain (*G*_10_) after 5 and 10 years compared to OCS-He* [**Figures 5B, C**; **Supplementary Material (Table S1 File S4)**]. Long-term commercial genetic gain (*G*_10_) after 60 years was also higher for UCPC-He* than for OCS-He* with He* = 0.01 in the 10 simulation replicates (on average, *G*_10_ = 22.869 [±0.641] compared to 21.892 [±0.525]) and less importantly with He* = 0.10 in nine out of 10 replicates (on average, *G*_10_ = 22.474 [±0.645] compared to 21.925 [±0.532]). However, for He* = 0.15, UCPC-He* outperformed OCS-He* at the long term in only three out of 10 replicates (on average, *G*_10_ = 20.665 [±0.573] compared to 20.938 [±0.553]) [**Figures 5B, C**; **Supplementary Material (Table S1 File S4)**]. The discounted cumulative gain giving more weight to short-term than to long-term gain (ρ = 0.04) was higher for UCPC-He* than OCS-He* in all simulation replicates for He* = 0.01 (on average, 12.321 [±0.284] compared to 11.675 [±0.262]), in all simulation replicates for He* = 0.10 (on average, 11.788 [±0.280] compared to 11.278 [±0.264]) and in nine out of 10 simulation replicates for He* = 0.15 (on average, 11.176 [±0.250] compared to 10.884 [±0.250]) (**Table 2**). Discounted cumulative gain giving the same weight to short- and long-term gain (ρ = 0) was also higher for UCPC-He* compared to OCS-He* (**Table 2**). When giving almost no weight to long-term gain after 30 years (ρ = 0.2), the best CSI appeared to be UC [on average, 6.822 (±0.145)] followed by the UCPC-He* with the lowest constraint on diversity (i.e., He* = 0.01) [on average, 6.682 (±0.143)].

**Table 2:** Discounted cumulative gain in TRUE scenario for three different parameters ρ giving more weight to short-term gain in different levels and assuming known QTL effects (TRUE scenario).

Cross-selection index (CSI)	Discounted cumulative gain		
	ρ = 0	ρ = 0.04	ρ = 0.2
UCPC - He* = 0.01	15.949 (±0.398)	12.321 (±0.284)	6.682 (±0.143)
UCPC - He* = 0.10	15.174 (±0.386)	11.788 (±0.280)	6.593 (±0.158)
UC	14.408 (±0.355)	11.689 (±0.266)	6.822 (±0.145)
OCS - He* = 0.01	15.148 (±0.346)	11.675 (±0.262)	6.360 (±0.149)
OCS - He* = 0.10	14.630 (±0.349)	11.278 (±0.264)	6.230 (±0.149)
UCPC - He* = 0.15	14.205 (±0.334)	11.176 (±0.250)	6.454 (±0.149)
OCS - He* = 0.15	14.056 (±0.337)	10.884 (±0.250)	6.103 (±0.155)
PM	12.609 (±0.280)	10.392 (±0.217)	6.345 (±0.155)

Mean discounted cumulative gain with ρ = 0 (constant weight along years), ρ = 0.04 (decreasing weight along years) and ρ = 0.2 (nearly null weights after 30 years) on the ten independent replicates. CSI are ordered in decreasing discounted cumulative gain with ρ = 0.04.

For a given He*, the additive genic variance ($\sigma_{a}^{2}$; **Figure 6A**) and genetic diversity at QTLs (He; **Figure 7A**) were constrained by the targeted diversity trajectory for both UCPC-He* or OCS-He*. However, UCPC-He* and OCS-He* behaved differently for genetic variance ($\sigma_{A}^{2}$; **Figure 6A**) resulting in differences for the ratio genetic over genic variances ($\sigma_{A}^{2}/\sigma_{a}^{2}$; **Figure 6C**). UCPC-He* yielded a higher ratio than OCS-He* (**Figure 6C**) independently of the targeted diversity He* at short term and midterm. For low targeted diversity (He* = 0.01), UCPC-He* showed in all 10 replicates a lower number of QTLs where the favorable allele was lost compared to OCS-He* (**Figure 7C**; **Supplementary Material [Table S1 File S4]**, on average 173.6 [±4.031] QTLs-194.3 [±2.633] QTLs).

#### GS Scenario With Estimated Marker Effects

**Figure 8:**
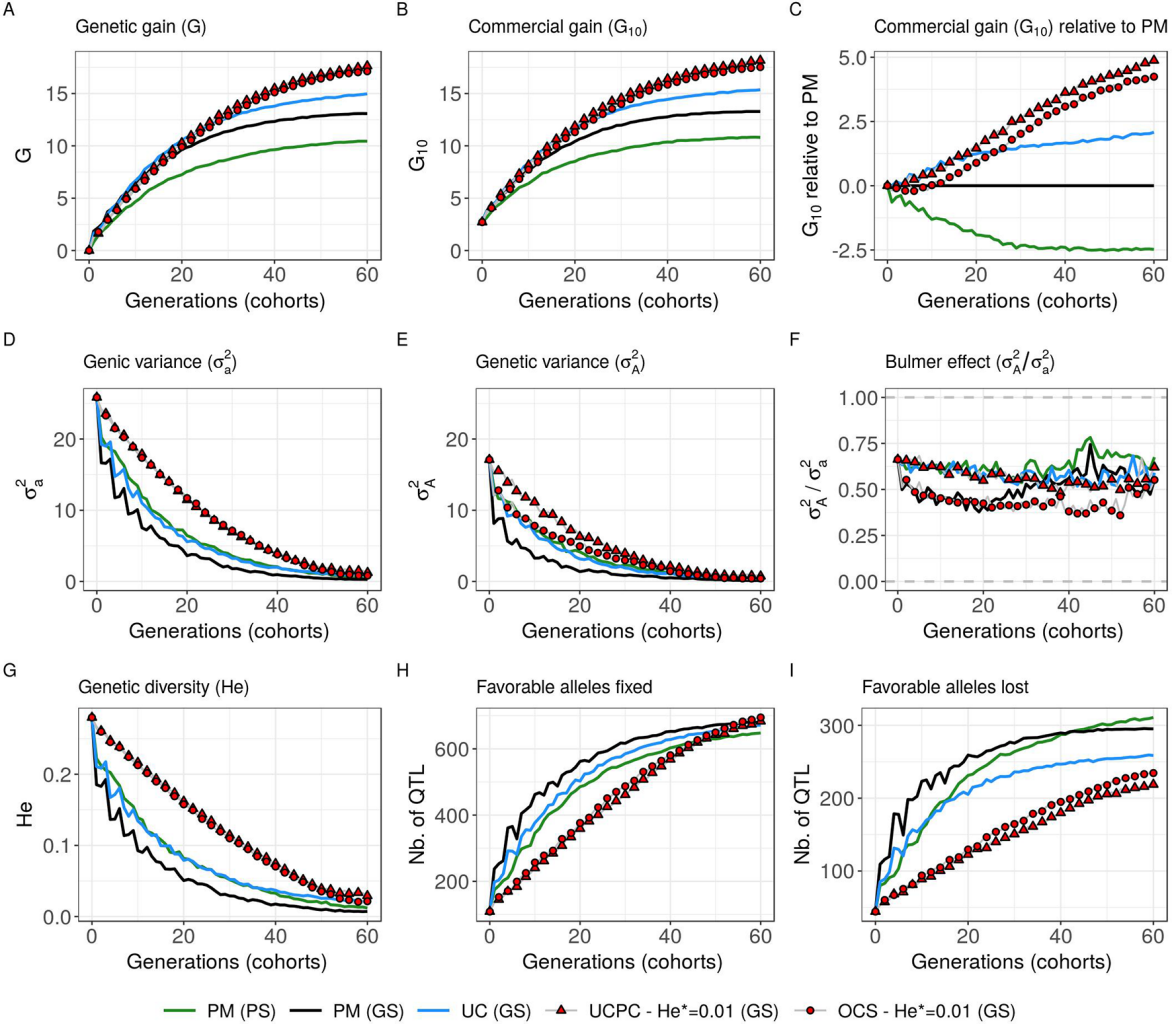
Evolution of different variables for different cross-selection indices according to the generations in the GS scenario (PM, parental mean; UC, usefulness criterion; OCS-He*, optimal cross-selection; and UCPC-He*, UCPC-based optimal cross-selection for He* = 0.01) and in the PS scenario (PM, parental mean). **(A)** Genetic gain at whole progeny level (G), **(B)** genetic gain at commercial level (*G*_10_), and **(C)** *G*_10_ relatively to PM (GS), genetic gain is measured on true breeding values. **(D)** Genic variance at QTLs ($\sigma_{a}^{2}$). **(E)** genetic variance of true breeding values ($\sigma_{A}^{2}$) and **(F)** ratio of genic over genetic variance ($\sigma_{A}^{2}/\sigma_{a}^{2}$). **(G)** genetic diversity at QTLs and number of QTLs where the favorable allele was fixed **(H)** and lost **(I)**.

Considering estimated marker effects (GS scenario) yielded lower genetic gain than when considering known marker effects [**Figures 5**–**8** and **Supplementary Material (Tables S1 and S2 File S4)**]. However, the short- and long-term superiority of the UC over the CSI ignoring within cross variance (PM) was consistent with estimated effects (on average, *G*_10_ = 8.338 [±0.237] compared to 7.713 [±0.256] 10 years post burn-in and *G*_10_ = 15.367 [±0.358] compared to 13.287 [±0.436] 60 years post burn-in; **Figure 8**, **Supplementary Material [Table S2 File S4]**). Similarly, the long-term superiority of UCPC-He* = 0.01 over UC was conserved in all 10 replicates (on average, *G*_10_ = 16.398 [±0.426] compared to 14.438 [±0.320] 40 years post burn-in and *G*_10_ = 18.161 [±0.470] compared to 15.367 [±0.358] 60 years post burn-in; **Figure 8**, **Supplementary Material [Table S2 File S4]**). Before the 40th year, UC and UCPC-He* = 0.01 performed similarly **Supplementary Material (Table S2 File S4)**. In GS scenario, UCPC-He* = 0.01 outperformed OCS-He* = 0.01 during the first 20 years in all 10 replicates (on average, *G*_10_ = 8.162 [±0.208] compared to 7.734 [±0.237] 10 years post burn-in and *G*_10_ = 11.881 [±0.272] compared to 11.313 [±0.323] 20 years post burn-in; **Figure 8**, **Supplementary Material [Table S2 File S4]**). After 20 years, UCPC-He* = 0.01 outperformed OCS-He* = 0.01 in eight out of 10 replicates (on average, *G*_10_ = 16.398 [±0.426] compared to 15.850 [±0.384] 40 years post burn-in and *G*_10_ = 18.161 [±0.470] compared to 17.528 [±0.438] 60 years post burn-in; **Figure 8**, **Supplementary Material [Table S2 File S4]**). Observations on the genic variance ($\sigma_{a}^{2}$ ) and genetic variance ($\sigma_{A}^{2}$) were consistent as well. We also observed that UCPC-He* = 0.01 yielded a lower number of QTLs where the favorable allele was lost (on average, 218.8 [±3.852]) compared to OCS-He* = 0.01 (on average, 234.5 [±3.908]) (**Figure 8**). PM not considering the marker information, i.e., phenotypic selection (PS scenario), yielded lower short- and long-term genetic gains than PM considering marker information (GS scenario) (on average, *G*_10_ = 6.402 [±0.166] compared to 7.713 [±0.256] 10 years post burn-in and *G*_10_ = 10.810 [±0.329] compared to 13.287 [±0.436) 60 years post burn-in; **Figure 8**, **Supplementary Material [Table S2 File S4]**).

## Discussion

### Predicting the Next-Generation Diversity

Accounting for within-family selection increased the squared correlation and reduced the mean error of post-selection genetic diversity prediction (**Figures 3**, **4**). The gain in squared correlation (**Figure 3**) and the reduction in mean error (**Figure 4**), were more important for parents showing differences in performance. This result is consistent with observations in Allier et al. (2019b), where crosses between two phenotypically distant parents yielded post-selection parental contributions that differ from their expectation before selection (i.e., 0.5). The mean prediction error was always positive, which can be explained by the use in Eq. 9 of genome-wide parental contributions to progeny in lieu of parental contributions at individual QTLs to predict allelic frequency changes due to selection **Supplementary Material (File S2)**. As a result, the predicted extreme frequencies at QTLs in the progeny are shrunk toward the mean frequency, leading to an overestimation of the expected heterozygosity (He) (results not shown). Local changes in allele frequency under artificial selection could be predicted following Falconer and Mackay (1996) and Gallais et al. (2007), but this approach would assume linkage equilibrium between QTLs, which is a strong assumption that does not correspond to the highly polygenic trait that we simulated.

### Effect of UC on Short- and Long-Term Recurrent Selection

In a first approach, we considered no constraint on diversity during cross-selection and compared cross-selection maximizing the UC or maximizing the PM in the TRUE scenario, assuming known QTL effects and positions. The UC yielded higher short-term genetic gain at commercial level (*G*_10_; **Figures 5B, C**). This was expected because UC predicts the mean performance of the best fraction of progeny. When considering the genetic gain at the mean progeny level (*G*; **Figure 5A**), UC needed 5 years to outperform PM. These results underline that UC maximizes the mean performance of the next generation issued from the intercross of selected progeny, sometimes at the expense of the current generation progeny mean performance. This observation is consistent with the fact that candidate parents of the sixth cohort came all from the three first cohorts generated considering UC and thus the sixth cohort took full advantage of the use of UC (**Figure 1A**). This tendency was also observed in simulations by Müller et al. (2018) considering the EMBV approach, akin to the UC for normally distributed additive traits. The UC also showed a higher long-term genetic gain at both commercial (*G*_10_) and whole progeny level (G) compared to intercrossing the best candidate parents (PM). This long-term gain was driven by a higher additive genic variance at QTLs ($\sigma_{a}^{2}$; **Figure 6A**) and a lower genomic covariance between QTLs ($\sigma_{A}^{2}/\sigma_{a}^{2}$; **Figure 6C**) resulting in a higher additive genetic variance in UC compared to PM ($\sigma_{A}^{2}$; **Figure 6B**). Note that with lower $\sigma_{a}^{2}$ the ratio $\sigma_{A}^{2}/\sigma_{a}^{2}$ becomes less interpretable in the long-term (**Figure 6C**). UC also better managed the fixation (**Figure 7B**) or the maintenance (**Figure 7C**) of the favorable allele at QTLs compared to PM. These results highlight the interest of considering within cross variance in cross-selection for improving long-term genetic gain as observed in Müller et al. (2018).

### Accounting for Within-Family Variance in Optimal Cross-Selection

Assuming known marker effects, we observed that considering a constraint on diversity, i.e., optimal cross-selection, always maximized the long-term genetic gain, at the cost of a variable penalty for short-term gain, compared to no constraint on diversity (e.g., UC). We further compared the OCS (Gorjanc et al., 2018) with the UCPC-based optimal cross-selection that accounts for the fact that only a selected fraction of each family contributes to the next generation. In the optimization framework considered, we compared the ability of UCPC (referred to as UCPC-He*) and OCS (referred to as OCS-He*) to convert a determined loss of diversity into genetic gain. For a given diversity trajectory, UCPC-He* yielded higher short-term commercial gain than OCS-He*. Both, OCS-He* and UCPC-He* yielded similar additive genic variance ($\sigma_{a}^{2}$), but we observed differences in terms of the ratio $\sigma_{A}^{2}/\sigma_{a}^{2}\;$. As expected under directional selection, the ratio $\sigma_{A}^{2}/\sigma_{a}^{2}$ was positive and inferior to one, revealing a negative genomic covariance between QTLs (Bulmer, 1971). UCPC-He* yielded a higher ratio, i.e., lower repulsion, and thus a higher additive genetic variance ($\sigma_{A}^{2}$) than OCS-He* for a similar He*. This explains the higher long-term genetic gain at commercial and whole progeny levels observed for UCPC-He*. This result supports the idea, suggested in Allier et al. (2019a), that accounting for complementarity between parents when defining crossing plans is an efficient way to favor recombination events to reveal part of the additive genic variance hidden by repulsion between QTLs. For low targeted diversity (He* = 0.01), UCPC-He* also appeared to better manage the rare favorable alleles at QTLs than OCS-He*. These results highlighted the interest of UCPC-based optimal cross-selection to convert the genetic diversity into genetic gain by maintaining more rare favorable alleles and limiting repulsion between QTLs. In case of higher targeted diversity (He* = 0.15), the loss of diversity was likely not sufficient to fully express the additional interest of UCPC compared to OCS to convert diversity into genetic gain. In this case, UCPC-He* and OCS-He* performed similarly. Accounting for within cross variance to measure the expected gain of a cross in optimal cross-selection was already suggested in Shepherd and Kinghorn (1998). More recently, Akdemir and Isidro-Sánchez (2016) and Akdemir et al. (2018) accounted for within cross variance considering linkage equilibrium between QTLs. Akdemir and Isidro-Sánchez (2016) also observed that accounting for within cross variance during cross-selection yielded higher long-term mean performance with a penalty at short-term mean progeny performance.

Short-term economic returns of a breeding program condition the resources invested to maintain/increase response to selection and therefore long-term competitive capacity. Hence, to fully take advantage of their benefit at long term, it is necessary to make sure that tested breeding strategies do not compromise too much the short-term commercial genetic gain. For this reason, we considered the discounted cumulative commercial gain following Dekkers et al. (1995) and Chakraborty et al. (2002) as a summary variable to evaluate CSI while giving more weight to short-term gain in different levels. UCPC-He* outperformed OCS-He* for a given He* either considering uniform weights (ဃρ = 0) or giving approximately seven times more weight to short-term gain compared to long-term gain (ρ = 0.04). This was also true when focusing only on short-term gain (ρ = 0.2), but in this case the best model was UC without accounting for diversity (**Table 2**).

### Practical Implementations in Breeding

#### UCPC With Estimated Marker Effects

In simulations, we first considered 1,000 QTLs with known additive effects sampled from a centered normal distribution. For a representative subset of CSIs (PM, UC, UCPC-He*, and OCS-He* with He* = 0.01; **Figure 8**), we considered estimated effects at 2,000 SNPs. The main conclusions obtained with known and estimated marker effects were consistent, supporting the practical interest of UCPC-based optimal cross-selection (**Figure 8**). The difference was that the superiority of UCPC-based optimal cross-selection over optimal cross-selection not accounting for within-family selection in GS scenario was not significant after 60 years **Supplementary Material (Table S2 File S4)**. With estimated marker effects instead of known QTL effects, the predicted progeny variance (σ^2^) corresponded to the variance of the predicted breeding values, which are shrunk compared to TBVs, depending on the model accuracy (referred to as variance of posterior mean [VPM] in Lehermeier et al.). An alternative would be to consider the marker effects estimated at each sample of a Monte Carlo Markov Chain process, e.g., using a Bayesian ridge regression, to obtain an improved estimate of the additive genetic variance (referred to as posterior mean variance [PMV] in Lehermeier et al., 2017a; Lehermeier et al., 2017b).

In practice, QTL effects are unknown, so the selection of progeny cannot be based on TBVs, and thus the selection accuracy (*h*) is smaller than one. In our simulation study assuming unknown QTLs (GS scenario), progeny were selected based on estimated breeding values taking into account genotypic information as well as replicated phenotypic information, which led to a high selection accuracy, as it can be encountered in breeding. Thus, the assumption *h* = 1 used in Eq. 6 for GS scenario is reasonable. In order to shorten the cycle length of the breeding scheme, selection of progeny can be based on predicted GEBVs of genotyped but not phenotyped progeny. In such a case, the selection accuracy (*h*) will be considerably reduced. In such a situation, one can advocate to use PMV instead of VPM in the computation of UCPC and to take into account the proper selection accuracy (*h*) within crosses adapted to the selection scheme. When selection is based on predicted values, i.e., genotyped but not phenotyped progeny, the shrunk predictor VPM should be a good approximation of (*h*σ)^2^.

#### UCPC-Based Optimal Cross-Selection 

In this study, we assumed fully homozygous parents and two-way crosses. However, neither the optimal cross-selection nor UCPC-based optimal cross-selection is restricted to homozygote parents. Considering heterozygote parents in optimal cross-selection is straightforward. Following the extension of UCPC to four-way crosses (Allier et al., 2019b), UCPC optimal cross-selection can be used for phased heterozygous individuals, as it is commonly the case in perennial plants or animal breeding. Animal breeders are interested in Mendelian sampling variance for individual and cross-selection (Segelke et al., 2014; Bonk et al., 2016; Bijma et al., 2018) and might be interested to incorporate it into OCS strategies. We considered an inbred line breeding program, but the extension to hybrid breeding is of interest for species such as maize. The use of testcross effects, i.e., estimated on hybrids obtained by crossing candidate lines with lines from the opposite heterotic pool, in UCPC-based optimal cross-selection is straightforward, and so the UCPC-based optimal cross-selection can be used to improve each heterotic pool individually. In order to jointly improve two pools, further investigations are required to include dominance effects in UCPC-based optimal cross-selection. In addition, this would imply that crossing plans in both pools are jointly optimized to manage genetic diversity within pools and complementarity between pools.

We considered a within-family selection intensity corresponding to the selection of the 5% most performant progeny as candidates for the next generation. Equal selection intensities were assumed for all families, but in practice due to experimental constraints or optimized resource allocation (e.g., generate more progeny for crosses showing high progeny variance but low progeny mean), within-family selection intensity can be variable. Different within-family selection intensities (see Eqs. 8 and 9) can be considered in UCPC-based optimal cross-selection, but an optimization regarding resource allocation of the number of crosses and the selection intensities within crosses calls for further investigations. However, in marker-assisted selection schemes based on QTL detection results (Bernardo et al., 2006), an optimization of selection intensities per family was observed to be only of moderate interest.

Proposed UCPC-based optimal cross-selection was compared to OCS in a targeted diversity trajectory context. We considered a linear trajectory, but any genetic diversity trajectory can be considered (e.g., **Figure 2**). The optimal diversity trajectory cannot be easily determined and depends on breeding objectives and data considered. Optimal contribution selection in animal breeding considers a similar ϵ-constraint optimization with a targeted inbreeding trajectory determined by a fixed annual rate of inbreeding (e.g., 1% advocated by the Food and Agriculture Organization (FAO), Woolliams et al., 1998). Woolliams et al. (2015) argued that the optimal inbreeding rate is also not straightforward to define. An alternative formulation of the optimization problem to avoid the use of a fixed constraint is to consider a weighted index (1-α)*V*(***nc***)+α*D*(***nc***), where α is the weight balancing the expected gain *V*(***nc***) and constraint *D*(***nc***) (De Beukelaer et al., 2017). However, the appropriate choice of α is difficult and is not explicit either in terms of expected diversity or expected gain.

#### Introgression of Diversity and Anticipation of a Changing Breeding Context

We considered candidate parents coming from the three last overlapping cohorts (**Figure 1**) in order to reduce the number of candidate crosses during the progeny covariances prediction (UCPC) and the optimization process. This yielded elite candidate parents that were not directly related (no parent–progeny) and that did not show strong differences in performances, which is standard in a commercial plant breeding program focusing on yield improvement. However, when the genetic diversity in a program is so low that long-term genetic gain is compromised, external genetic resources need to be introgressed by crosses with internal elite parents. As suggested by results of simulation 1, we conjecture that the advantage of UCPC-based optimal cross-selection over OCS increases in such a context where heterogeneous, i.e., phenotypically distant, genetic materials are crossed. This requires investigations that we hope to address in subsequent research.

Our simulations also assumed fixed environments and a single targeted trait over 60 years. However, in a climate change context and with rapidly evolving societal demands for sustainable agricultural practices, environments and breeders objectives will likely change over time. In a multitrait context, the multiobjective optimization framework proposed in Akdemir et al. (2018) can be adapted to UCPC-based optimal cross-selection. The upcoming but yet unknown breeding objectives make the necessity to manage genetic diversity even more important than highlighted in this study.

## Data Availability Statement

Publicly available datasets were analyzed in this study. This data can be found here: https://doi.org/10.25387/g3.7405892.

## Author Contributions

ST, CL, AC, and LM supervised the study. AA performed the simulations and wrote the manuscript. ST worked on the implementation in the simulator. All authors reviewed and approved the manuscript.

## Funding

This research was funded by RAGT2n and the ANRT CIFRE grant no. 2016/1281 for AA.

## Conflict of Interest

The authors declare that the research was conducted in the absence of any commercial or financial relationships that could be construed as a potential conflict of interest.
