# Supplementary material for: Improving Short- and Long-Term Genetic Gain by Accounting for Within-Family Variance in Optimal Cross-Selection
Source: Front Genet. 2019 Oct 29;10:1006. doi: 10.3389/fgene.2019.01006 (PMC6828944; doi:10.3389/fgene.2019.01006)
Supplement: Supplementary file 2 [file DataSheet_2.pdf]

Improving short and long term genetic gain by accounting for within family variance in optimal cross selection

Antoine Allier[12*], Christina Lehermeier[2], Alain Charcosset[1], Laurence Moreau[1], Simon Teyssèdre[2]

[1] GQE - Le Moulon, INRA, Univ. Paris-Sud, CNRS, AgroParisTech, Université Paris-Saclay, 91190 Gif-sur-Yvette, France

[2] RAGT2n, Genetics and Analytics Unit, 12510 Druelle, France

[*] **Correspondence:** Antoine Allier (antoine.allier@inra.fr)

File S2

Relationship between IBS coancestry and genetic diversity in progeny

The identity by state (IBS) coancestry between N inbred parents is defined as:

$$\boldsymbol{K} = 0.5\left(\frac{1}{m}\left(\boldsymbol{X}\boldsymbol{X}'\right) + \mathbf{1}_N\mathbf{1}_N'\right), \text{ (Eq. 1)}$$

where, $\boldsymbol{X}$ is the genotyping matrix of the N parents in line and m loci in column, with elements coded -1 or 1 and $\mathbf{1}_N$ is a N-dimensional column vector of ones.

Considering the N-dimensional column vector of expected parental genomewide contributions $\boldsymbol{c}$, with $c(j),\ j \in [1, N]$ the contribution of the parent j to progeny, the mean expected IBS coancestry in progeny is:

$$IBS = \boldsymbol{c}'\boldsymbol{K}\,\boldsymbol{c} = 0.5\left[\frac{1}{m}\left(\boldsymbol{c}'\boldsymbol{X}\boldsymbol{X}'\boldsymbol{c}\right) + \boldsymbol{c}'\mathbf{1}_N\mathbf{1}_N'\boldsymbol{c}\right]. \text{ (Eq. 2a)}$$

Note that $\boldsymbol{c}'\mathbf{1}_N\mathbf{1}_N'\boldsymbol{c} = 1$ since $\sum_{j=1}^{N}\boldsymbol{c}(j) = 1$. Then, Eq. 2a simplifies:

$$IBS = 0.5\left[\frac{1}{m}\left(\boldsymbol{c}'\boldsymbol{X}\boldsymbol{X}'\boldsymbol{c}\right) + 1\right] \text{ (Eq. 2b)}$$

The mean expected genetic diversity (He) in progeny is:

$$He = \frac{1}{m}\,\mathbf{1}_m'\left(2\,\boldsymbol{p}\circ\left(\mathbf{1}_m - \boldsymbol{p}\right)\right), \text{ (Eq. 3a)}$$

where $\mathbf{1}_m$ is a m-dimensional column vector of ones, $\circ$ is the pairwise entry product and $\boldsymbol{p}$ is the m-dimensional column vector of expected allelic frequencies in progeny:

$$\boldsymbol{p} = 0.5\ \left(\left(\boldsymbol{X} + \mathbf{1}_N\mathbf{1}_m'\right)' \circ \mathbf{C}\right)\mathbf{1}_N, \text{ (Eq. 4a)}$$

where $\mathbf{C}$ is the $(m \times N)$-dimensional matrix of expected local parental contributions to progeny with $C(i,j),\ i \in [1, m],\ j \in [1, N]$ the contribution of parent j to progeny at the locus i. $C(i,j), \forall\, i \in [1, m]$

is further approximated by the genomewide parental contribution to progeny $c(j)$. Consequently, the m-dimensional column vector of expected allelic frequencies (Eq. 4a) is approximated as:

$$\tilde{\boldsymbol{p}} = 0.5\ (\boldsymbol{X} + \mathbf{1}_N \mathbf{1}'_m\ )'\boldsymbol{c}. \text{ (Eq. 4b)}$$

We replace $\boldsymbol{p}$ by its approximation $\tilde{\boldsymbol{p}}$ in Eq. 3a:

$$\widetilde{He} = \frac{1}{m}\ \mathbf{1}'_m \left((\boldsymbol{X}'\boldsymbol{c} + \mathbf{1}_m\mathbf{1}'_N\boldsymbol{c}) \circ (\mathbf{1}_m - 0.5\ \boldsymbol{X}'\boldsymbol{c} - 0.5\ \mathbf{1}_m\mathbf{1}'_N\boldsymbol{c})\right). \text{ (Eq. 5a)}$$

Note that $\mathbf{1}_m\mathbf{1}'_N\boldsymbol{c} = \mathbf{1}_m$ and Eq. 5a becomes:

$$\widetilde{He} = \frac{1}{m}\ \mathbf{1}'_m \left((\boldsymbol{X}'\boldsymbol{c} + \mathbf{1}_m) \circ (0.5\ \mathbf{1}_m - 0.5\ \boldsymbol{X}'\boldsymbol{c})\right)$$

$$= \frac{1}{m}\ \mathbf{1}'_m \left(0.5\ (\mathbf{1}_m - \boldsymbol{X}'\boldsymbol{c} \circ \boldsymbol{X}'\boldsymbol{c})\right)$$

$$= 0.5\ \left(1 - \frac{1}{m}\ \mathbf{1}'_m\ (\boldsymbol{X}'\boldsymbol{c} \circ \boldsymbol{X}'\boldsymbol{c})\right). \text{ (Eq. 5b)}$$

Let us note $\boldsymbol{v} = \boldsymbol{X}'\boldsymbol{c}$. It can be shown that $\mathbf{1}'_m\ (\boldsymbol{v} \circ \boldsymbol{v}) =\ \boldsymbol{v}'\boldsymbol{v}$, resulting in:

$$\widetilde{He} = 0.5\ \left(1 - \frac{1}{m}\ (\boldsymbol{c}'\boldsymbol{X}\boldsymbol{X}'\boldsymbol{c})\right) = 0.5(1 - 2\ IBS + 1) = 1 - IBS. \text{ (Eq. 6)}$$

Note that this equivalence is conserved whether we consider ante- or post-selection parental contributions ($\boldsymbol{c}$), respectively in OCS or in UCPC based OCS.
