# Supplementary material for: Improving Short- and Long-Term Genetic Gain by Accounting for Within-Family Variance in Optimal Cross-Selection
Source: Front Genet. 2019 Oct 29;10:1006. doi: 10.3389/fgene.2019.01006 (PMC6828944; doi:10.3389/fgene.2019.01006)
Supplement: Supplementary file 4 [file DataSheet_4.pdf]

Improving short and long term genetic gain by accounting for within family variance in optimal cross selection

Antoine Allier[12*], Christina Lehermeier[2], Alain Charcosset[1], Laurence Moreau[1], Simon Teyssèdre[2]

[1] GQE - Le Moulon, INRA, Univ. Paris-Sud, CNRS, AgroParisTech, Université Paris-Saclay, 91190 Gif-sur-Yvette, France

[2] RAGT2n, Genetics and Analytics Unit, 12510 Druelle, France

[*] **Correspondence:** Antoine Allier (antoine.allier@inra.fr)

File S4:

Supplementary tables

Table S1 TRUE scenario: Mean commercial genetic gain (G_{10}) and genetic gain (G) at different generations (5 years, 10 years, 20 years, 40 years and 60 years) and mean number of QTLs where the favorable allele has been lost at after 60 years. In brackets is given the standard error divided by the square root of number of independent replicates ($\sqrt{10}$).

CSI	Commercial genetic gain (G_{10})					Genetic gain (G)					# of QTL where the favorable allele is lost after 60 years
	5 years	10 years	20 years	40 years	60 years	5 years	10 years	20 years	40 years	60 years	
**PM (TRUE scenario)**	6.184 (+/- 0.174)	8.338 (+/- 0.195)	11.861 (+/- 0.280)	15.118 (+/- 0.373)	15.744 (+/- 0.449)	4.647 (+/- 0.174)	7.197 (+/- 0.199)	11.085 (+/- 0.258)	14.869 (+/- 0.353)	15.735 (+/- 0.447)	274.9 (+/- 4.283)
**UC (TRUE scenario)**	6.574 (+/- 0.170)	9.316 (+/- 0.208)	13.369 (+/- 0.316)	17.553 (+/- 0.460)	18.293 (+/- 0.516)	4.633 (+/- 0.138)	7.620 (+/- 0.158)	12.290 (+/- 0.286)	17.139 (+/- 0.441)	18.280 (+/- 0.513)	243.1 (+/- 4.547)
**OCS - He*=0.01 (TRUE scenario)**	5.924 (+/- 0.130)	8.563 (+/- 0.224)	12.743 (+/- 0.294)	18.821 (+/- 0.447)	21.892 (+/- 0.525)	3.918 (+/- 0.133)	6.810 (+/- 0.187)	11.326 (+/- 0.277)	18.017 (+/- 0.429)	21.656 (+/- 0.529)	194.3 (+/- 2.633)
**UCPC - He*=0.01 (TRUE scenario)**	6.317 (+/- 0.139)	9.164 (+/- 0.201)	13.550 (+/- 0.322)	19.752 (+/- 0.538)	22.869 (+/- 0.641)	4.024 (+/- 0.120)	7.018 (+/- 0.149)	11.859 (+/- 0.285)	18.832 (+/- 0.507)	22.626 (+/- 0.634)	173.6 (+/- 4.031)
**OCS - He*=0.10 (TRUE scenario)**	5.901 (+/- 0.136)	8.455 (+/- 0.193)	12.239 (+/- 0.310)	17.872 (+/- 0.445)	21.925 (+/- 0.532)	3.838 (+/- 0.119)	6.490 (+/- 0.183)	10.547 (+/- 0.276)	16.739 (+/- 0.411)	21.237 (+/- 0.507)	110.7 (+/- 3.768)
**UCPC - He*=0.10 (TRUE scenario)**	6.327 (+/- 0.175)	8.927 (+/- 0.198)	12.972 (+/- 0.326)	18.475 (+/- 0.510)	22.474 (+/- 0.645)	3.915 (+/- 0.129)	6.760 (+/- 0.187)	11.051 (+/- 0.294)	17.178 (+/- 0.471)	21.643 (+/- 0.621)	109.7 (+/- 3.876)
**OCS - He*=0.15 (TRUE scenario)**	5.785 (+/- 0.161)	8.118 (+/- 0.211)	11.800 (+/- 0.276)	17.148 (+/- 0.422)	20.938 (+/- 0.553)	3.708 (+/- 0.144)	6.185 (+/- 0.182)	10.042 (+/- 0.255)	15.747 (+/- 0.390)	19.867 (+/- 0.525)	87.9 (+/- 4.365)
**UCPC - He*=0.15 (TRUE scenario)**	6.215 (+/- 0.186)	8.643 (+/- 0.194)	12.248 (+/- 0.311)	17.187 (+/- 0.439)	20.665 (+/- 0.573)	3.803 (+/- 0.132)	6.402 (+/- 0.186)	10.246 (+/- 0.252)	15.670 (+/- 0.389)	19.528 (+/- 0.546)	90.3 (+/- 5.439)

Table S2 GS and PS scenarios: Mean commercial genetic gain (G_{10}) and genetic gain (G) at different generations (5 years, 10 years, 20 years, 40 years and 60 years) and mean number of QTLs where the favorable allele has been lost at after 60 years. In brackets is given the standard error divided by the square root of number of independent replicates ($\sqrt{10}$).

CSI	Commercial genetic gain (G_{10})					Genetic gain (G)					# of QTL where the favorable allele is lost after 60 years
	5 years	10 years	20 years	40 years	60 years	5 years	10 years	20 years	40 years	60 years	
**PM (PS scenario)**	4.925 (+/- 0.165)	6.402 (+/- 0.166)	8.507 (+/- 0.270)	10.371 (+/- 0.343)	10.810 (+/- 0.329)	2.827 (+/- 0.156)	4.672 (+/- 0.154)	7.241 (+/- 0.222)	9.633 (+/- 0.339)	10.445 (+/- 0.318)	310.8 (+/- 4.250)
**PM (GS scenario)**	5.543 (+/- 0.198)	7.713 (+/- 0.256)	10.423 (+/- 0.331)	12.769 (+/- 0.414)	13.287 (+/- 0.436)	4.013 (+/- 0.175)	6.509 (+/- 0.170)	9.655 (+/- 0.326)	12.326 (+/- 0.403)	13.084 (+/- 0.427)	295.2 (+/- 3.708)
**UC (GS scenario)**	5.984 (+/- 0.211)	8.338 (+/- 0.237)	11.660 (+/- 0.314)	14.438 (+/- 0.320)	15.367 (+/- 0.358)	4.088 (+/- 0.174)	6.672 (+/- 0.226)	10.530 (+/- 0.285)	13.790 (+/- 0.311)	14.971 (+/- 0.336)	258.6 (+/- 4.571)
**OCS - He*=0.01 (GS scenario)**	5.546 (+/- 0.198)	7.734 (+/- 0.237)	11.313 (+/- 0.323)	15.850 (+/- 0.384)	17.528 (+/- 0.438)	3.418 (+/- 0.154)	5.894 (+/- 0.191)	9.896 (+/- 0.309)	15.114 (+/- 0.369)	17.128 (+/- 0.429)	234.5 (+/- 3.908)
**UCPC - He*=0.01 (GS scenario)**	5.930 (+/- 0.179)	8.162 (+/- 0.208)	11.881 (+/- 0.272)	16.398 (+/- 0.426)	18.161 (+/- 0.470)	3.544 (+/- 0.129)	6.049 (+/- 0.178)	10.290 (+/- 0.252)	15.486 (+/- 0.388)	17.633 (+/- 0.465)	218.8 (+/- 3.852)
